# Highlights from the WIN 2017 Symposium, 26–27 June 2017, Paris, France: ‘Expediting Global Innovation in Precision Cancer Medicine’

**DOI:** 10.3332/ecancer.2017.770

**Published:** 2017-09-27

**Authors:** Will Davies

**Affiliations:** Cancer Intelligence, 13 King Square Avenue, Bristol, BS2 8HU, UK

**Keywords:** personalised medicine, genomics, biomarkers, targeted therapy, sequencing

## Abstract

The Worldwide Innovative Networking (WIN) symposium brings together representatives from academic institutions, pharmaceutical partners, technology companies and charitable organisations from across the globe for an annual summit, discussing ongoing research and the latest developments in precision medicine. Now, in its seventh year, the aims of the WIN consortium’s annual meeting, to foster communication and collaboration between members and deliver clinical trial results that improve the care and outcomes of patients are presented in open dialogue to encourage debate and discussion.

This year, the meeting was held in Paris, France from 26–27 June and consisted of six plenary sessions, two debates, and poster presentations from attendees. In keeping with the consortium’s goals, presentations and posters focused on the development and integration of new therapies and updates in genome-based medicine.

Among the presentations at this year’s meeting, much of the focus fell on design and implementation of new designs of clinical trials, moving away from decades-long assessments of thousands of patients towards a nimble, adaptive design fitting the edicts of personalised medicine and delving into greater depths within genomic data, ranging beyond genome analysis to chart new targets in ligandomics, proteogenomics and more.

This year’s symposium was opened with a cheerful welcome from WIN chairman Dr John Mendelsohn (MD Anderson Cancer Centre, Houston, TX, USA), who had much reason to speak warmly to the audience. In the intervening year between this symposium and the last, more and more targeted drugs have entered clinical trials as monotherapy and combinations, more still have reached US/European approval, and the concept of genomic sequencing is even reaching the lay public. Looking forward from this point, Dr Mendelsohn offered a short wish list for technical and social support for personalised medicine platforms and practitioners. Among them were as follows:

- diagnostic platforms that can support treatment decisions, providing a summary of detected biomarkers, suitable trials to which the patient may be recruited and response rates to current care,
- the integration of Big Data into the patient’s own journey through disease onset, diagnosis, treatment and aftercare, as well as taking that data forward to inform and improve outcomes for subsequent patients,
- biomarkers beyond genes alone, and making full use of transcriptomics, proteomics and molecular imaging to give the most refined, up-to-date and wholly personalised view of a patient’s biology in the moment that they are diagnosed and treated.

It is no small order to turn a capacity-led healthcare system on its head to pour tremendous resources and enormous cost into the absolute understanding of a momentary glimpse of a single tumour specimen. But these wishes are perhaps not so far away, as the next two days made clear.

The first to take on the challenge of delivering an answer to these high ideals was Dr José Baselga (Memorial Sloan Kettering Cancer Center, New York, USA), in his keynote lecture on genomic-driven clinical studies in breast cancer. Breast cancer benefits from years of focus and development in identifying biomarkers for disease subtypes and risk, with survival rates rapidly improving in a single generation. With an eye to the future, Dr Baselga set out his own forecast on implementing genome-driven oncology. It handily tacks close to Dr Mendelsohn’s own dreams, and Dr Baselga illustrated his ideal process of samples arriving from solid and liquid biopsies for sequencing, analysis to guide selection of targeted therapy, which is deployed in adaptive combinations, and reacting to markers for evolution or disease progression through ongoing sample surveillance. To offer some current context, he also introduced phase-I studies of PI3Kinase inhibitor taselisib, which is now escalating to a phase-III trial named SANDPIPER in combination with fulvestrant. This combination, he hopes, will improve specificity and reduce toxicity for breast cancer patients. AKT inhibitors were another treatment option discussed, as monotherapy or in combination with immunotherapy, which is where many see personalised therapy as offering the most promise.

The first plenary session opened with a theme that defined the rest of the day: innovation in clinical trial design. The first two presentations came courtesy of Dr Laura Esserman (University of California San Francisco, USA) and Dr Donald A. Berry (MD Anderson Cancer Center, Houston, TX, USA), two of the leads behind the I-SPY2 trial for breast cancer. I-SPY2 is notable for its adaptive randomisation technique and 12 experimental arms.

Berry built on this trial format in setting out how he sees the course of trial design evolving to match pace with new understandings of cancer biology; progression from ‘learning’ in adaptive phase-I studies to ‘confirming’ in phase II ought to be a smooth flow, with identified markers or subtypes driving adaptiveness and with re-randomisation open to patients.

Similar thoughts were echoed by Dr J. Jack Lee (MD Anderson Center, Houston, TX, USA), who gave a mathematical twist to designs with his presentation on Bayesian adaptive designs. Much as in the I-SPY2 trial, Lee endorses moving trials of personalised therapies towards smaller, focused phase-III trials from adaptive arms. Given the very nature of personalised medicine, the scale and rigidity of traditional trials recruiting thousands of patients seems ill matched to countering a continually evolving disease.

These aspects of cancer were characterised by Dr Razelle Kurzrock (University of California, San Diego Moores Cancer Center, USA) as ‘malignant snowflakes’, each metastatic tumour being unique and requiring equally nimble therapy combinations to match. Taking example from new paradigms in treating CML, Dr Kurzrock advocated being ready to bring treatments to earlier stages in the course of disease development, edging up towards the cytotoxic combinations discussed on day 2 by Prof René Bernards.

Much of the hopes and guidance Dr Kurzrock had previously outlined were discussed further in the subsequent debate session on trends in clinical trial design. Here, she clearly articulated a counter to concerns of personalised therapies disappearing into a rabbit hole of subtyping when treating disease—of course patients are individuals, but the strategy applies to all of them. Drafting a global strategy suitable for differing healthcare providers proved further contentious, with concerns of next-generation sequencing used *post hoc* as an expensive confirmation of disease relapse. For others concerned about the costs in terms of economic and human outcomes, the long game of disease management remained a priority.

Immunotherapy, of course, is proving effective in treating solid tumours, with PD1 antibodies rarely out of the headlines since their success and subsequent approval for first-line treatment of metastatic NSCLC from ESMO 2016. So, when Dr Patrice Denèfle (Institute Roche for Research & Translational Medicine, France) opened proceedings of the second plenary session, it is with little surprise that he highlighted immune-based combinations and new tactics in his talk on integrating data into drug and biomarker discovery. Beyond pointing out that there are over 800 ongoing immunotherapy combination trials, volume and scale were key points in Dr Denèfles’ presentation.

He spoke of raising a T-cell army with tumour vaccines, of one target in therapy being not enough in treatment regimens, of too much information being generated from genome networks for any single brain too hold. Solutions to these challenges being at the core of the WIN consortium’s mission—combined therapies and open, integrated genomic databases, has Dr Denèfle preaching to the choir. The onus is now on the audience to, as Denefle says, ‘Do now what patients need next’.

On the subject of what comes next, the field of personalised oncology would be loath to rest on its laurels. Sign posts for research, which may come to take the world by storm could be found across the rest of the session, with Dr Geneviève Almouzni (Institut Curie, France) outlining the steps in replication regulation that might open treatment pathways through their cellular chaperones, histone variants which function as ‘architects of chromatin organisation’, and epigenetic changes in patients, down to their non-coding RNA. The path she wove from chromatin as a functional molecule in space and time to impacts on signalling and epigenetic expression illustrated the shifting backdrop of a living human cell with fascinating mechanistic insights, even without the context of wider roles in tissues and organs. If, as Dr Almouzni promised, epigenetics is to be most active area of drug development, the simple approach to personalised therapy of treating mutations found in a patient’s tumour may have to take on new layers of complexity.

Similarly, Dr Giorgio Massimini (Merck KgaA, Germany) reported a hot topic in cancer drug design—the DNA damage-response pathway. With new components showing promise as druggable targets, and a growing catalogue of success in prostate and ovarian cancer with olaparib PARP inhibition, Dr Massimini’s view from the helm of phase-I trials for ATR kinase inhibitors as monotherapies and in combination is a welcome addition to a growing arsenal.

The third plenary session, immunological approach to personalised medicine, tapped in to the aforementioned triumphs of immune modulatory agents and outlines ongoing work to anchor that success in new trials. Unsurprisingly combinations come to the fore, as in Prof Antoni Ribas’ (University of California Los Angeles, USA) talk on new biomarkers for response to immunotherapy. With ongoing discussions of the use of surrogate biomarkers, the utility of liquid biopsy for sample collection, and Prof Ribas’ proposed combinations of BRAF, MEK and anti-PD1 therapy for melanoma, there seems little room for any tumour cells escaping.

This total encapsulation of tumour biology and behavior was only furthered by Dr Roy Baynes (MSD, USA), who also examined the enrolment of patients and architecture of collaborative research groups. In his session on histology-agnostic development of immune oncology agents, Dr Baynes reported on the immunogenic potential of cancer cells with hampered DNA damage repair, as identified via microsatellite instability assays, meaning patients may be suitable for pembrolizumab monotherapy and enrolment in combination trials. In regards to challenges faced in enrolling patients to multihistology enrichment basket studies, Dr Baynes noted a barrier to patient enrolment of diagnostic test positivity was a requirement, as was the enrolment of a ‘reasonable numbers’ of patients. This dovetailed neatly into the earlier debates of clinical trial construction, with some favouring the smaller, nimble adaptive trials, as did Dr Baynes’ position that cancer centres may do well to organise clinics not by tumour type, but by biomarker status in the future.

Patients and positivity remained centre stage with the last presenter of this session, Dr Steve Anderson (COVANCE, USA), who took the baton of patient enrolment and spoke on the challenges of stratifying patients for immunotherapy. Within the context of research conducted at COVANCE as a clinical research organisation, Dr Anderson highlighted many of the biomarkers for target and response identified by Prof Ribas, above, including neo-antigen burden, genomic instability and markers down to a patient’s history of infection in their virome as means for sorting and strategising patient approaches when treating with immune therapies. Looking back at Dr Mendelsohn’s wish list from the morning, you would be hard pressed to find any deeper integration of biomarker data.

Personalised medicine does not all take place at the microlevel though, with the patient and their well-being existing in much larger scale. Prof Guido Kroemer’s (Gustave Roussy, France) presentation took macro-level treatment of disease very much to heart (or rather stomach) as he gave fascinating insights into apoptotic pathways that can influence treatment response. Chief among these was the impact of fasting states on immune-independent autophagy, which could be mimicked with protein deacytelating cis-regulatory molecules. Proofs of concept here came by examining the changes in metabolomes of mouse models and human volunteers, including Prof Kroemer himself.

The final presentation of Monday returned to the molecular level but offered a promise of population wide delivery of personalised therapies as Prof Hans-Georg Rammensee (University of Tübingen, Germany) spoke about patient-specific peptide vaccines. Delving again into the proteome, Rammensee began with identifying tumour specific antigens, cancer-bound peptides believed to be highly immunogenic but often sequestered, which may attract and bind to circulating T cells. This ligandome, as he describes it, may hold the key to designing personal cancer vaccines, tailored on a patient-to-patient basis to make the most of detected mutations and amplify immune responses, with a turnaround of weeks from initial tumour resection. Between the use of adjuvant therapies and immune-modulation to further prime the tumour microenvironment and sharpen the senses of circulating T cells, it might almost be all of Dr Mendelsohn’s dreams come true at once. Anchoring this potential to proven clinical results and achieving regulatory approval remains a step in need of taking, but if ever there was an audience able to take up that challenge, a WIN symposium would be where to find them.

The next hour was given over to a second debate considering the place of immunotherapy approaches in precision cancer care.

Beginning with one of the major stories from recent months, the first topic for debate was the difference in reported outcomes for pembrolizumab versus nivolumab in lung cancer, with pembrolizumab approved for first-line treatment, whereas nivolumab (submitted for approval in a broader patient population) was not. Antoni Ribas, one of the debate moderators, noted the potential for observation bias in non-responding patients being potentially hyper-progressors and emphasised that understanding non-response is as important as pursuing patients who do respond.

The conversation then turned to one of construction, considering the different forms of antibodies and whether their design, inclusive of an FC region or not, may affect clinical performance and macrophage interference. The comparison was made of avelumab, a monoclonal antibody with an FC region to bind IgG1, compared to atezolizumab and durvalumab, which lack such activity. Of all these PD-L1-targeted therapies, avelumab seems to be the most well tolerated, most specific, and yield the most durable response [1], so FC incorporation may be key to future therapy structures.

Future therapies are where the conversation, inevitably, turned. The consortium’s focus on combination therapies surfaced again, with discussion on inter-company co-operation in testing and trial design, though, as the moderators were sure to point out, those clinical trials will require pre-clinical evidence of treatment schedule, dosing, and use in a humanised model. A counterpoint was made to take the previously suggested modifications to trial execution forward to the earliest stages—generate a small trial for the agent or combination in question, sequence patient samples before, during and after to observe responses and figure out what worked where, for whom, and why, in a *post hoc* fashion. Such investigative biology, for lack of a better term, would require radical changes in treatment and trial design, regulation, interpretation and incorporation into wider health strategy.

Special thanks were given to the winners for best poster presentation, Vivek Subbiah (MD Anderson Cancer Center, Houston, TX, USA) for his research on combining vandetanib and everolimus in RET-rearranged lung cancer, and Prof Ui-Soon Koon (University of Hong Kong, Hong Kong) for her investigation of a biomarker for tamoxifen-resistant breast cancer.

Sessions resumed the following morning with the 4^th^ plenary topic of ‘Next great steps in cancer therapy’, and a return to the omic-focused content that could well define this conference, and the future of oncology. First, Olli Kallioniemi (Karolinska Institutet, Stockholm, Sweden) discussed novel models for personalising cancer therapies and delivering once again on Mendelsohn’s wish of using genomic data as guidance in trials. Pointing to the comparative ease of liquid biopsy to guide treatment choices in leukaemia, he highlighted the success of axitinib to treat relapse BCR Abl+ disease after the onset of resistance.

However, heightened mutational understanding of acute myeloid leukaemia has not been matched by increased survival prospects, leading Kallioniemi to introduce his Individual Systems Medicine. The flow of data here is simple; from sample gathering through sensitivity testing, biobanking and profiling down to the signalome, to generate results that are acted on in treatment or trial selection, during which further samples are gathered. To prove the point further, he illustrated the sequencing data from patients, which has revealed expected, and unexpected, clustering of treatment efficacies. Drugs of the same families offered predictable synergy, but sequencing of select patients data revealed vulnerabilities to combinations, which have not yet been clinically assessed in larger populations.

So ought these patients be eligible for exploratory treatments with a novel combination as an n-of-1 trial? As the debate panel from Monday noted, hurdles in approval, regulation and reimbursement of such a scheme persist.

Speaking of which, Henry Rodriguez (National Cancer Institute, USA) took the stage next to offer a summary of the CPTAC programs of the National Institute of Health, which have so far seen ~2000 users accessing almost 300TB of proteogenomics data, in a completely open and free fashion. Rodriguez also introduced the APOLLO program (apparently named independently of the Beau Biden Moonshot Initiative) to routinely sequence patients and screen for targeted therapy opportunities. Between the global collaboration, open data and integration of genomic screening into therapy selection, Dr Mendelsohn must be very happy to have had so many items on his opening wish list addressed by the end of the same symposium.

Sequencing and selection of therapy was similarly discussed by Peter Lichter (DKFZ, Germany), with a focus on targeting mutations in the Sonic Hedgehog gene family, which he has found to be driving paediatric medulloblastomas, with Smoothin inhibitors, and using algorithmic assessments of disease predisposition. Among medulloblastoma subgroups, he also noted the chance of enhancer hijacking, in which a tumourigenic gene alteration is translocated to be downstream of enhancement regions, causing significant acceleration of disease. Current research is at the level of animal models to determine site-specific inhibition for these cases, but the future patients afflicted by these rarer aggressive subtypes is looking brighter already.

Stefan Fröhling (DKFZ, Germany) gave further insight, relating the design and initial outcomes of the NCT MASTER platform (Molecularly Aided Stratification for Tumour Eradication Research). Again, sample processing led to genome and RNA sequencing led to analysis and evaluation, eventually sorting patients into different intervention groups. This then funnels into the INFORM group for paediatric patients, discussed by Stefan Pfister later, and the MASTER MATCH groups for rare adult tumours.

Martine Piccart (Institute Jules Bordet, Belgium), next to speak, gave a comprehensive review of past and current work to determine biomarkers for breast cancer, opening with a summary of the APHINITY trial that ‘we won’t do better for HER2 disease’. So, given the tremendous improvement in survival curves over the last 15 years, she talked instead of damage control and reduction, highlighting the CLEOPATRA trial of added docetaxel as improving progression free survival, and the PANACEA trial now bringing PD1 antibodies into play. Another novel combination comes in the form of the ZEPHIR trial, utilising zircon-labelled trastuzumab with PET/CT scanning to pre-emptively screen for patients unlikely to benefit from further trastuzumab emtansine. In her overview, Piccart repeatedly emphasised the view of patients at the heart of trials and treatment regimens—‘Patients don’t want pathologic complete responses, they want to be alive. Complete responses can relapse’. She also noted that, for efforts in staging treatments as neo-adjuvant or adjuvant interventions, ‘In neoadjuvant, we have markers. None have shown predictive use in adjuvant settings’. What we are left with then is not precision medicine, she said, but stratified choices; making sure that any patient gets their best treatment.

The next two speakers gave alternate views on the development and application of personalised therapies: Dr Jean-François Martini (Pfizer Inc., USA) presented the industry view of attempting to discern and design drugs against clinically relevant biomarkers, including the development of S-Trac assay for sunitinib, but it was the work of Vanessa Michelini (IBM Watson Health, USA) that caught the attention and imagination of the audience. IBM Watson Health is subset of the broader IBM Watson framework, in which databases are fed thousands of research papers, building a vast library of interconnected data. When presented with a patient’s biomarker profile, Watson Health returns a suggested course of treatment, based on literature available. The obvious hyperbole of computers replacing doctors was quickly dismissed, though the platform’s 90% concordance with physician’s choices for treating breast cancer, as presented at the San Antonio Breast Cancer Symposium in 2016 [2], was touted as a promising indicator for a future in which a quorum of options and opinions can quickly give a treatment of best fit. Given the wealth of genomic data currently being generated, and conference attendees’ clearly stated goals of adding further depth and breadth of information captured, such a platform seems to be the only way to handle the challenge.

The last presentation of the fifth plenary session came courtesy of Prof Caroline Robert (Institut Gustave Roussy, Paris, France), touching on two conference themes in her research on combination therapies with an immune-based component. In this case, the combination was with tyrosine kinase inhibitors to treat melanoma. Checkpoint therapies and BRAF/MEK-targeted therapies have shown tremendous gains for patient survival and recovery in isolation, and Prof Robert related results from pre-clinical trials on novel combination schedules to stay one step ahead of resistance development. As TKIs enter a new clinical generation, and those working on checkpoint inhibitors look to either broaden their targets or sharpen the edge of response curves, the prospect of both working in tandem seems an exciting one, and worth keeping a close eye on between now and WIN 2018 for trial expansion.

Prof René Bernards (NKI, the Netherlands) opened the final session with his work on selecting drug combinations based on synthetic lethality. The one-two punch theory of staggered synergy seeks to keep tumours from developing a secure niche and resistance. He made the case for treating BRAF melanomas that have acquired MEK resistance with histone deacetylases—the metabolic compensation of tumour cells leaves them uniquely susceptible to the toxic levels of reactive oxygen species generated, whereas healthy host tissue remains intact. Taking this to a more broadly applicable strategy, Prof Bernards described a possible opportunity in selectively inducing senescence in cancer cells as the opening salvo, followed by a senolytic chaser to expunge all traces of cancer regardless of any one resistance pathway. Cell culture and animal models are currently in trials and have so far shown an exciting possibility for repurposed sertraline; based on results of an experimental molecule XL413 that induces senescence in hepatocellular carcinoma cells, the Zoloft generic seems to selectively induce apoptosis. More on this as it develops.

The research of Dr Daniel F. Hayes (University of Michigan Comprehensive Cancer Center, Ann Arbor, USA) lent itself very handily to audience interests as well, focusing on the incorporation of serial liquid biopsies into trials for selection and surveillance of patients and responses. Seeing as this has been on the wish list and idealised work flow of several prior presenters, any steps towards its practical application were welcome.

Dr Reinhard Büttner (University Hospital of Cologne, Cologne, Germany) spoke further on determining vulnerabilities in tumours, in this case through the Network Genomic Medicine framework in Germany. Given his focus on lung cancer, the past years watershed success of PD1-targeted therapies cannot be avoided, and he outlined how capture-based sequencing and profiling for PD-L1 can be delivered with point-of-access pathology for rapid results. Dr Büttner also took the story of cancer development back to its beginnings—looking for germline or acquired mutations to establish risk, limiting environmental exposure, and encouraging early detection, much as in Dr Leroy Hoods P4 platform, below.

Next, Dr Stefan Pfister (DKFZ, Germany) picked up research first touched upon by Stefan Fröhling and Peter Lichter, reporting on the INFORM (INdividualised therapy For relapsed Malignancies in childhood and adolescence) program for personalised, population-based paediatric oncology. A rare cancer overall, paediatric brain tumours are most lethal in their age group, and Dr Pfister described how the sequencing of samples from children across 50 cancer centres is delivering rapid, rational targeting options and contrasts these results to standard chemotherapy. While these results were an expected expansion following Dr Pfister’s 2014 introduction of INFORM, the changing state of sequencing availability and circulating sample biopsy places them in interesting context and may serve as a useful framework for future interventions.

The final presentation of Robert G. Bristow (Princess Margaret Cancer Centre, Canada) looked at prognosis and prospects for prostate cancer patients in an age of targeted therapy. With prostate cancer still the most common malignancy in men worldwide, Bristow offered new insights into the genomic hallmarks of different disease types. He described the genomic rearrangements separating metastatic castration-resistant prostate cancer from sporadic disease, and a characteristic methylome. Bristow went on to introduce the concept of a ‘nimbosus’ in prostate cancer cells—a gathering of intraductal carcinoma cells with cribiform architecture (IDC/CA+) associated with genomic instability, metastatic spread and high lethality. Between these and BRCA/Wnt-mutant cells posing a challenge requiring intensive therapies, he advised finding treatment targets outside of the shifting sea of mutational targets for comparatively simple tumours. As for what shape those targets take, there remain many other -omics yet to explore.

Those thoughts led neatly to the last discussion session of the symposium, a roundtable session considering the challenges of implementing precision medicine and what that means for other diseases. Already, the FDA has approved ivacaftor as a precision medicine for cystic fibrosis [3].

Participants in this session provided a broad-ranging discussion of the challenges in implementation of cancer precision medicine with a focus on identifying treatment combinations likely to be uniquely effective in individual patients and strategies to manage the high cost of cancer care that is likely to be further exacerbated with the use of expensive drugs in combination.

## Conclusion

With the concluding remarks of Leroy Hood (Institute for Systems Biology, USA) again exhorting P4 systems medicine and scientific wellness, a long-view attitude to health that comes somewhere between sociology and engineering in which a healthy lifestyle with regular omni-omic surveillance can reveal cancer precursors long before a patient would normally seek treatment, the symposium drew to a close. Where last year’s conference looked to a future of immunotherapy and combinations, this year saw Dr Mendelsohn’s opening wish list met with ready answers and expansion in mind. The future is coming, fast. Ready or not.
